# Genetic Origin of Osseous Metaplasia of the Endometrium: A Case Series

**DOI:** 10.7759/cureus.80145

**Published:** 2025-03-06

**Authors:** Mona Mishra, Neetu Singh, Rupita Kulshrestha

**Affiliations:** 1 Obstetrics and Gynecology, Dr. Ram Manohar Lohia Institute of Medical Sciences, Lucknow, IND

**Keywords:** bone in the endometrium, endometrial polymerase chain reaction, endometrium, genetic analysis, osseous, osseous metaplasia

## Abstract

Osseous metaplasia of the endometrium is the presence of mature and immature bone tissue inside the endometrial cavity. We present a case series of three women who presented with various complaints of vaginal discharge and menstrual abnormalities. On hysteroscopy, bone fragments were extracted. Histopathological study of the bone tissue was supportive of osseous metaplasia. We performed a DNA analysis of the bone and compared it to the maternal genotype. We found a complete match between the patient and bone genotype, thus supporting that the bone originates from the patient.

## Introduction

True osseous metaplasia is the presence of mature and immature bone tissue inside the endometrial cavity. It most frequently occurs during the reproductive years, but it has been reported in post-menopausal women also [1]. Women present with infertility and a variety of symptoms like menometrorrhagia, vaginal discharge, and dyspareunia. Osseous metaplasia of the endometrium is rare with an incidence of 3/10,000, and less than 100 cases have been reported until now [2]. The current literature is uncertain regarding the genetic origin of osseous metaplasia. Various theories have been hypothesized regarding its etiology, but the most common theory is the metaplastic transformation of endometrial stromal cells into osteoblasts [3]. It has been falsely attributed to the retention of fetal bones inside the uterine cavity after a prior abortion. Contrary to the belief that it has fetal origin, osseous metaplasia is genetically of maternal origin. This can be demonstrated by comparing the patient's short tandem repeats (STR) and STR of the bone tissue. We present a case series of three cases, out of which in one case we performed DNA analysis of the bone and compared it with the maternal genotype. The study revealed that the DNA profile of the bone sample showed a 100% match with the maternal DNA profile, thus confirming that the bone sample is of maternal origin rather than fetal origin.

## Case presentation

Case 1

**Figure 1:**
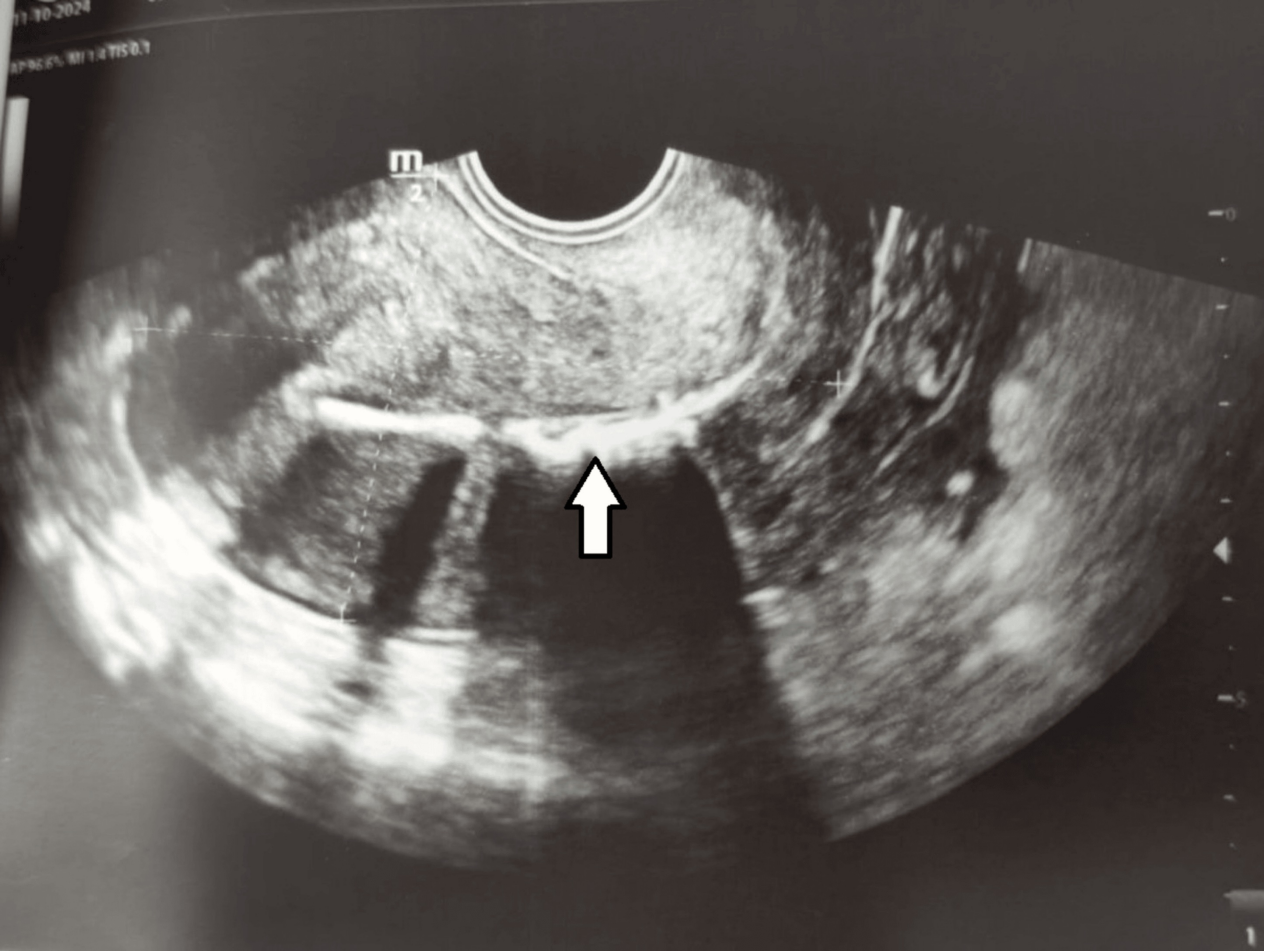
2D transvaginal ultrasound reveals multiple echogenic structures inside the endometrial cavity, the largest measuring 17.5 mm (white arrow).

A 35-year-old woman presented to the outpatient clinic with the complaint of irregular vaginal spotting associated with vaginal discharge. She was P3L3A2 with a history of surgical abortion twice previously. Two years back, she underwent a first-trimester surgical abortion. When she presented to us, there was no history of menstrual overdue, and the urine pregnancy test was negative. Her examination findings were unremarkable. Her 2D transvaginal ultrasound revealed multiple echogenic structures inside the endometrial cavity, the largest measuring 17.5 mm (Figure 1).

**Figure 2:**
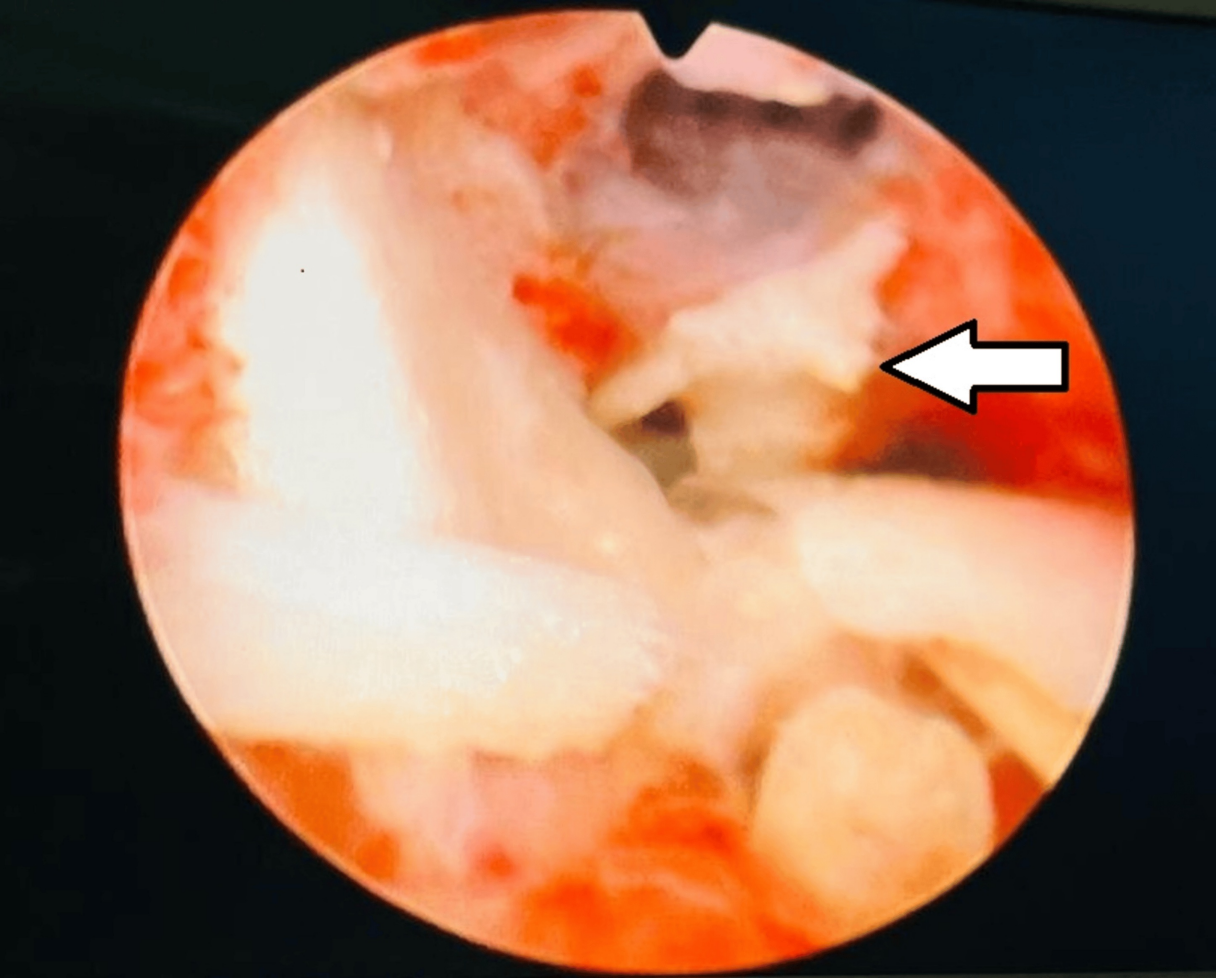
On hysteroscopic view, multiple bone-like structures are seen inside the uterine cavity (white arrow).

**Figure 3:**
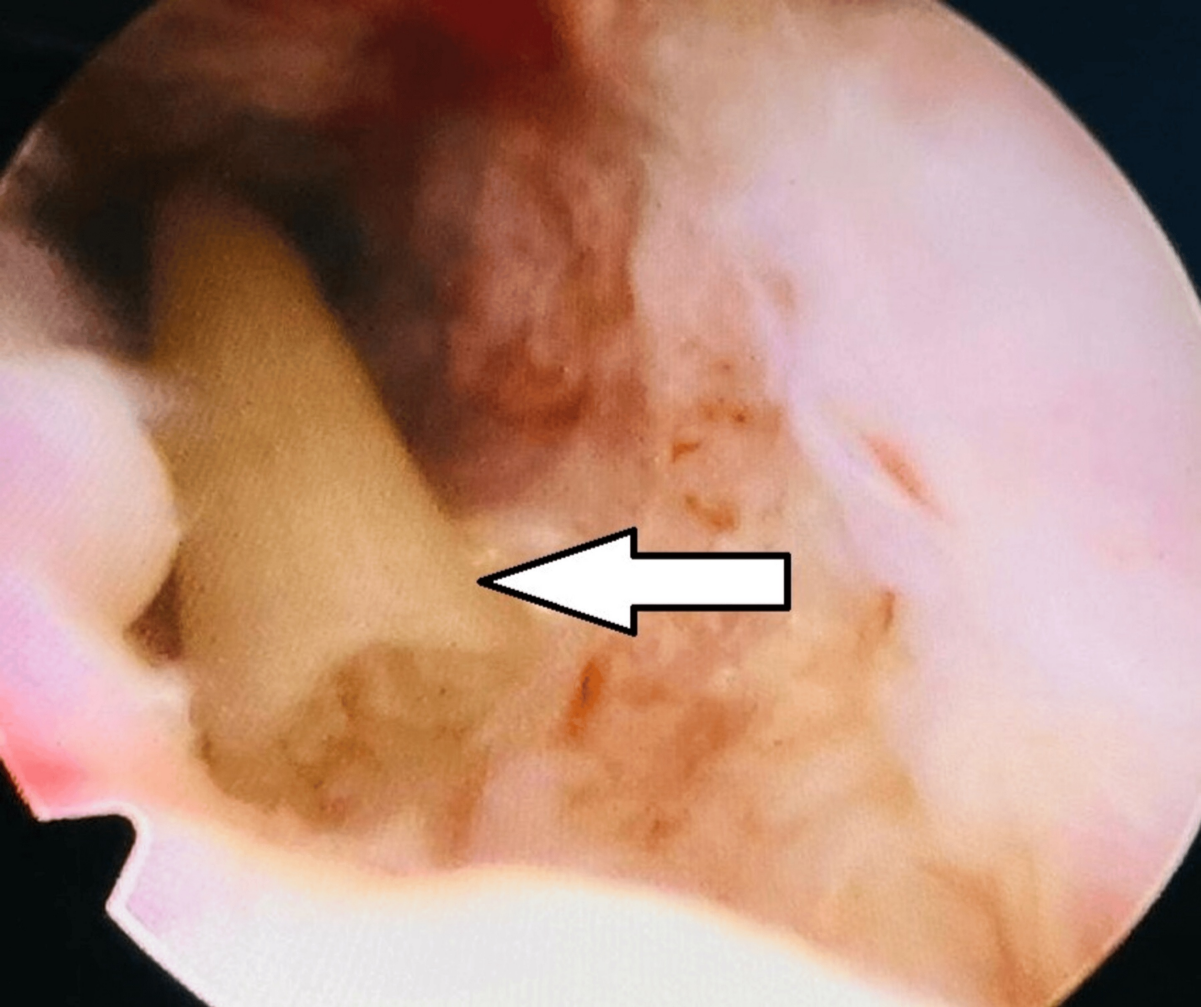
A long bone structure is seen inside the uterine cavity on hysteroscopy (white arrow).

A diagnostic hysteroscopy revealed multiple bone-like structures inside the uterine cavity (Figure 2 and Figure 3).

**Figure 4:**
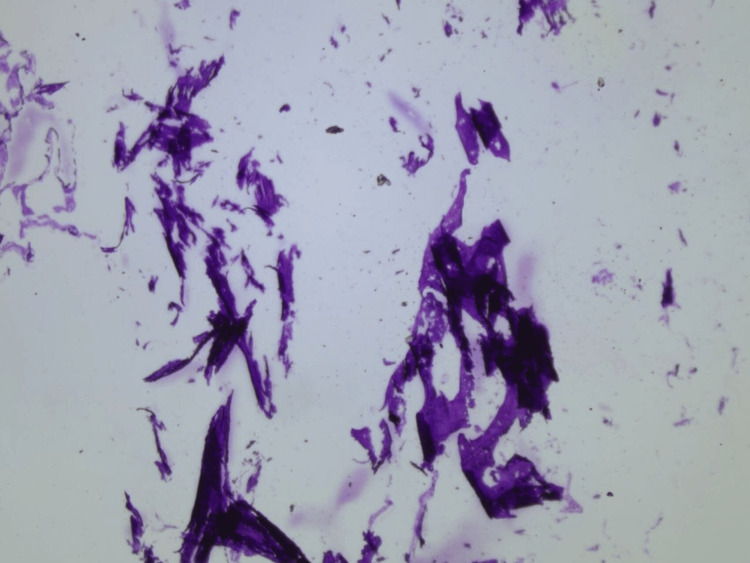
Photomicrograph demonstrates predominantly calcified tissue and endometrial tissue ossification (4× magnification) on hematoxylin and eosin staining.

We removed the bone fragments with graspers inserted inside the operating channel of the rigid hysteroscope. The bone fragments were sent for histopathology and DNA analysis. Histopathology of the bone fragment was consistent with osseous metaplasia (Figure 4).

The patient's sample and the bone sample were sent for DNA analysis, to compare both the genotypes.

Procedure for DNA collection, polymerase chain reaction (PCR) amplification, and analysis

DNA Collection

Bone marrow specimens were processed under highly sterile conditions to preserve nucleic acid integrity and prevent contamination. Samples were aliquoted into 1.5 mL microcentrifuge tubes and subjected to proteolytic digestion by adding 20 µL of Proteinase K, 200 µL of lysis buffer containing chaotropic agents, 200 µL of phosphate-buffered saline (PBS) to maintain isotonicity, and 50 µL of sodium dodecyl sulfate (SDS) to enhance membrane disruption and protein denaturation. The samples were incubated at 56°C for one hour to facilitate the enzymatic degradation of proteins and then at 80°C to ensure complete tissue lysis and the denaturation of residual proteins or enzymes.

Following digestion, genomic DNA was precipitated by adding 200 µL of 100% ethanol to the lysate, promoting DNA binding to the silica-based membrane during subsequent column purification. The lysate was passed through the silica column 3-4 times to maximize DNA adsorption, followed by sequential washing steps to remove contaminants, including residual salts and degraded proteins. Finally, the purified DNA was eluted in 30 µL of AE buffer. DNA extraction was conducted using the QIAamp DNA Mini Kit (QIAGEN, Hilden, Germany), adhering to the manufacturer's protocols for optimal yield and purity.

PCR Amplification

Quantitative fluorescence polymerase chain reaction (QF-PCR) assays were established using the Devyser Extend v2 Kit (Årsta, Sweden), which includes primers for amplifying specific chromosome loci. The assays utilized fluorescently labeled primers targeting STR markers across chromosomes X, Y, 21, 18, and 13. STR markers, which exhibit polymorphic variations in the number of repeat units, were employed as genetic markers for DNA sequence identification and the verification of chromosomal dosage.

Extracted DNA from the bone marrow was diluted to a working concentration of 20 ng/µL using Qubit fluorometry for quantification (Thermo Fisher Scientific, Waltham, Massachusetts, United States). For each sample, 5 µL of DNA was added to 20 µL of the master mix provided in the kit. PCR amplification was performed in a final volume of 25 µL. The thermal cycling conditions included an initial denaturation at 95°C for 15 minutes, followed by 27 cycles of denaturation at 94°C for 30 seconds, annealing at 58°C for one minute and 30 seconds, and extension at 72°C for one minute and 30 seconds. A final extension step at 72°C for 30 minutes was carried out, and the reaction was held at 4°C indefinitely.

Capillary Electrophoresis

Post-amplification, the PCR products were analyzed using capillary electrophoresis. A loading cocktail was prepared by mixing 2 µL of the size standard (e.g., 560 SIZER ORANGE) with 100 µL Hi-Di Formamide (Thermo Fisher Scientific, Waltham, Massachusetts, United States). From this mix, 15 µL was dispensed into each well of a microwell plate. Subsequently, 1.5 µL of each PCR product was added to the corresponding wells. The plate was sealed and loaded onto the ABI Genetic Analyzer (Applied Biosystems, Waltham, Massachusetts, United States) for separation.

Data Interpretation

Analysis of the fluorescence signals was conducted using GeneMapper v3.7 software (Applied Biosystems, Waltham, Massachusetts, United States). STR markers were identified and quantified by calculating the peak area ratios of alleles. Informative markers demonstrated heterozygosity with two distinct peaks of equal intensity, corresponding to alleles of different lengths. The peak area ratios were used to evaluate chromosomal dosage and detect potential aneuploidies. Results interpretation adhered to QF-PCR principles, accurately identifying chromosomal abnormalities based on the differential amplification of STR markers.

Result of QF-PCR

**Figure 5:**
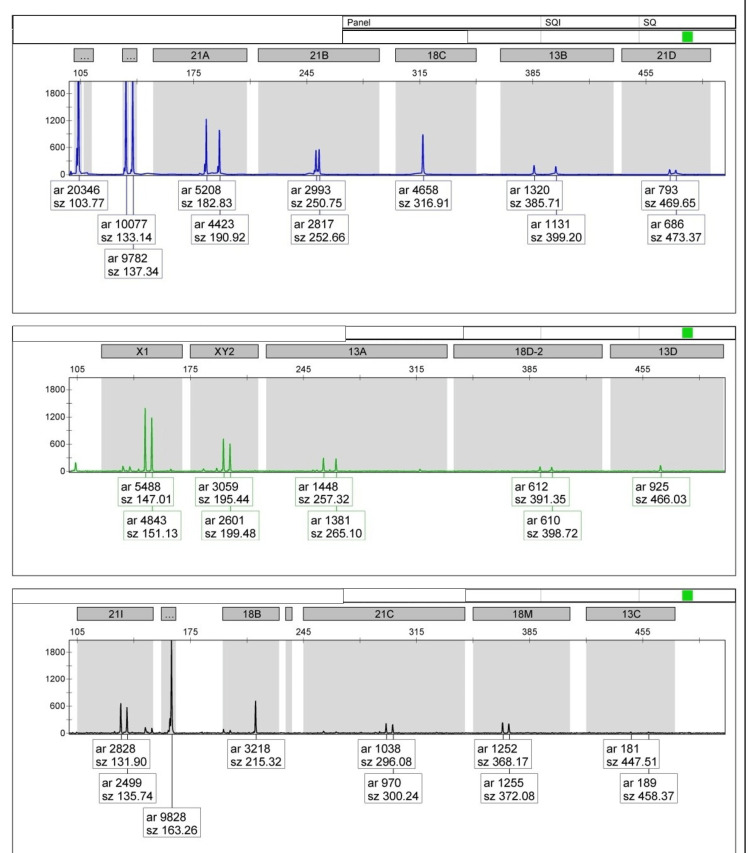
QF-PCR shows the same allele size detected in the maternal peripheral blood and endometrial bone sample. QF-PCR: quantitative fluorescence polymerase chain reaction

QF-PCR showed the same allele size detected in maternal peripheral blood and endometrial bone samples, suggesting identical alleles. The analysis suggested shared genetic material, supporting the evidence of maternal contribution to the endometrial tissue (Figure 5).

Case 2

**Figure 6:**
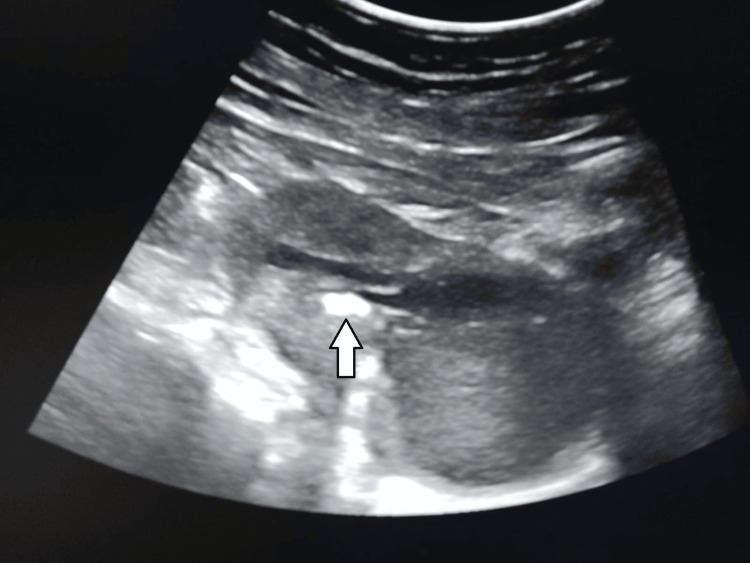
2D transvaginal ultrasound shows a large collection inside the uterine cavity with homogenous internal echoes and echogenic foci (white arrow).

A 35-year-old nulliparous woman with primary infertility presented with secondary amenorrhea for two months. She had no previous history of cervical or intrauterine procedures. She had an episode of heavy menstrual bleeding six months back. Apart from this episode, her past menstrual cycles were regular with average flow. She was evaluated at a private healthcare facility, and an echogenic structure in her endometrial cavity was seen on transvaginal ultrasound. Suspecting a foreign body, the echogenic structure was extracted under general anesthesia. Histopathology of the echogenic structure revealed stratified squamous metaplasia with bone formation. Six months later when she presented to our facility with secondary amenorrhea, her abdomen was soft and non-tender. On speculum examination, the cervix was unremarkable with vaginal discharge. Bimanual examination revealed a non-tender bulky uterus and free adnexa. Her 2D transvaginal ultrasound revealed a large intrauterine collection with homogenous internal echoes and echogenic foci in the endometrial cavity (Figure 6). Echogenic heterogeneous dependent contents were also noted in the collection.

**Figure 7:**
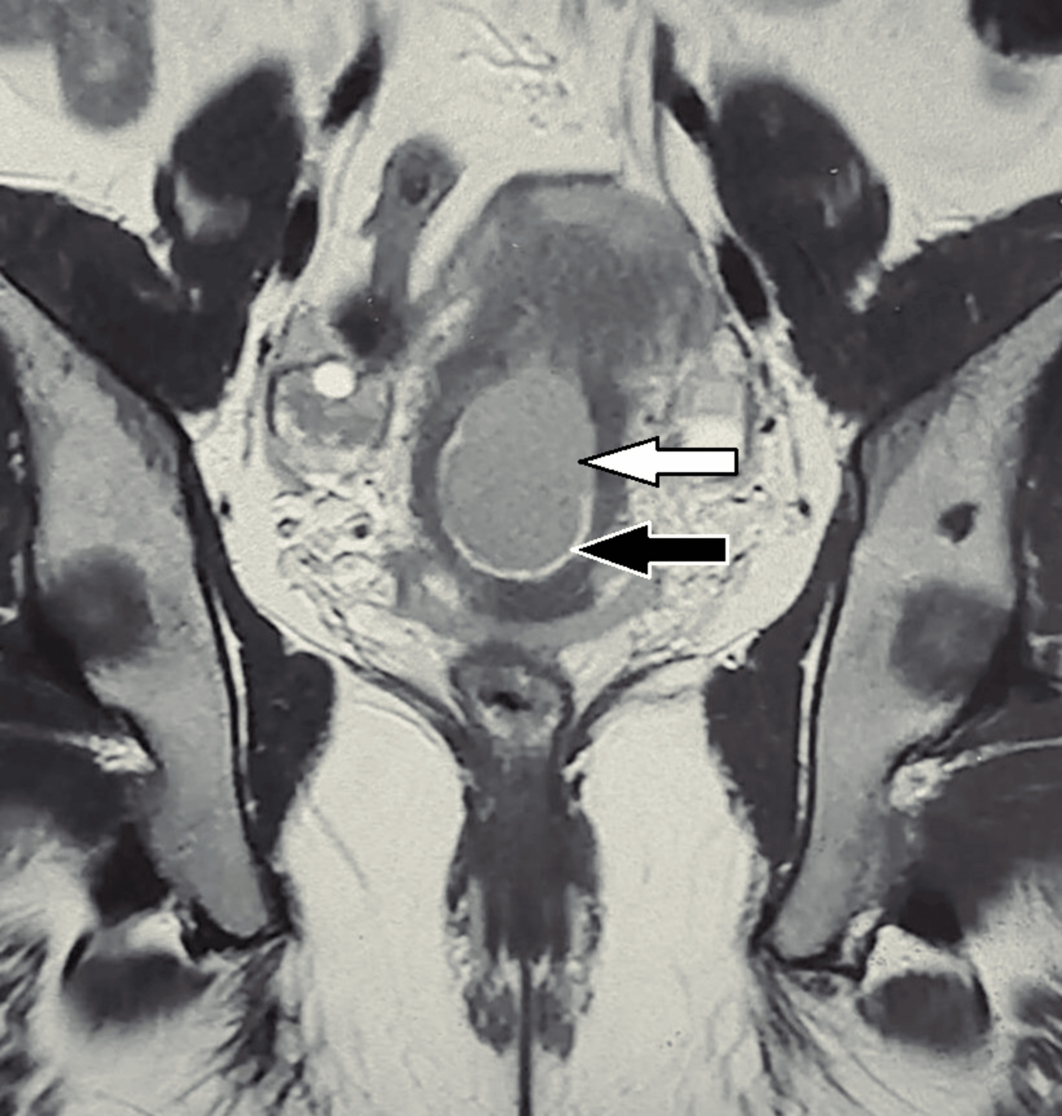
T2-weighted coronal MRI shows a hyperintense to intermediate signal intensity lesion in the lower uterine segment, endometrial cavity, and endocervical canal (black arrow). Evidence of hemorrhagic collection is seen inside the uterine cavity (white arrow). MRI: magnetic resonance imaging

Magnetic resonance imaging (MRI) showed a bulky uterus with well-defined T1 and T2/short tau inversion recovery (STIR) hyperintense to intermediate signal intensity lesions in the lower uterine segment, endometrial cavity, and endocervical canal. Blooming was seen on gradient echo (GRE) images suggestive of a hemorrhagic collection inside the uterine cavity. There was no evidence of diffuse restriction. Endocervical stroma and the endometrium appeared to maintain signal intensity (Figure 7).

**Figure 8:**
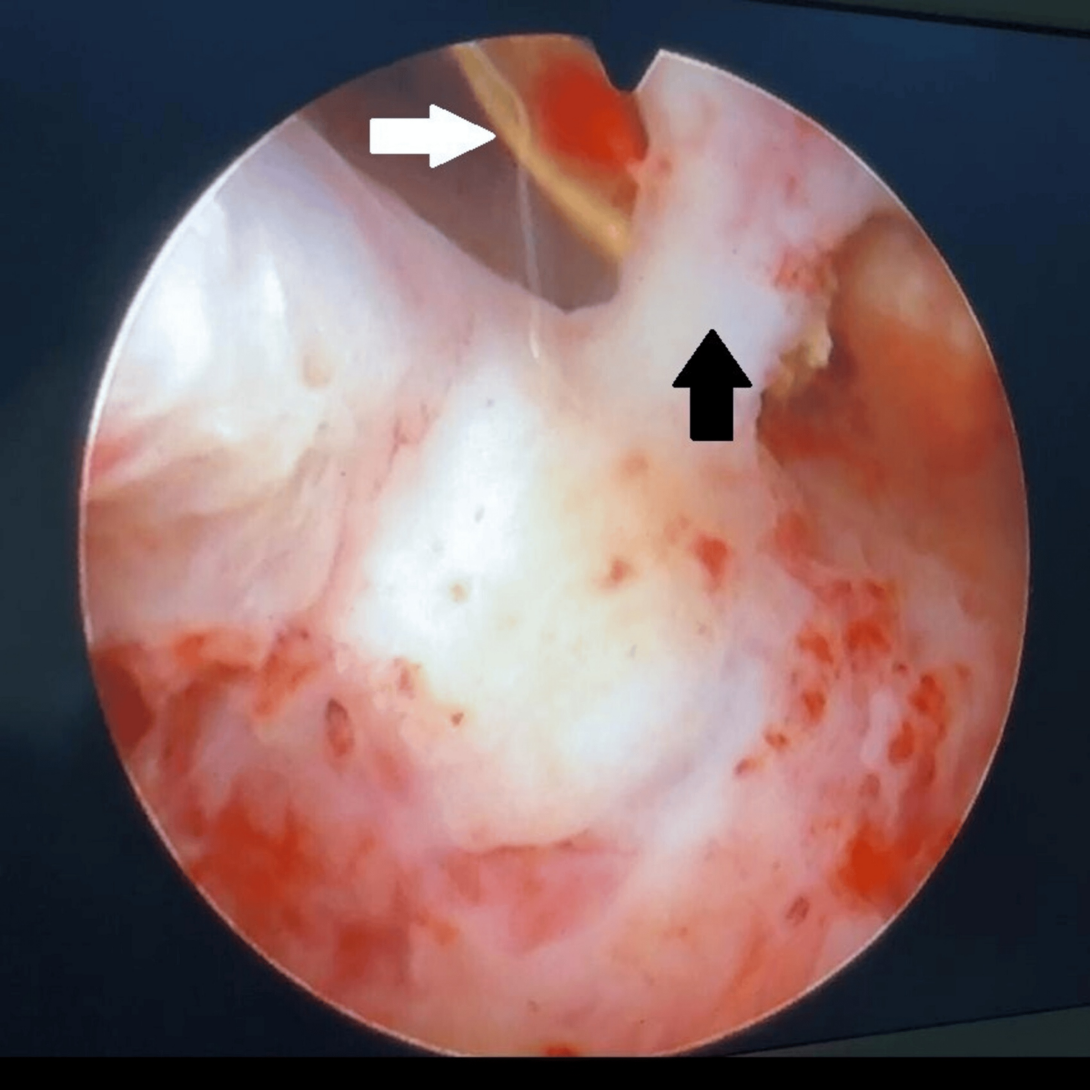
A hysteroscopic view of the bone fragment (white arrow) and the synechiae (black arrow) in the uterine cavity.

We performed a diagnostic hysteroscopy on the patient. After progressive dilatation of the cervix, around 100 ml of altered blood was evacuated from the uterine cavity. Hysteroscopy showed multiple trabecular bony fragments in the endometrial cavity (Figure 8).

Fragments were removed with graspers inserted in the operating channel of a rigid hysteroscope. Multiple synechiae in the uterine cavity were seen which were lysed with scissors. A tissue biopsy of endometrial tissue was obtained and analyzed for histopathology, Ziehl-Neelsen (ZN) stain, and Mycobacteria Growth Indicator Tube (MGIT) culture. A size 8 pediatric catheter was inserted in the uterine cavity immediately after hysteroscopy which was removed after 10 days. We gave oral estrogen treatment to prevent the reformation of adhesions postoperatively along with timed progestin therapy to induce withdrawal bleeding. Histopathological evaluation showed tissue comprising round-oval endometrial glands with tall columnar epithelium with minimal intervening stroma showing fibrosis with osseous metaplasia. Histological evaluation of the surrounding endometrium in our case showed evidence of mild inflammatory infiltrate and intervening stromal fibrosis. No acid-fast bacilli were found in the endometrial tissue on the ZN stain, and the MGIT culture was sterile.

After the procedure, the patient started having regular menses. She was well, asymptomatic, and reviewed in the preconception clinic to embark on pregnancy.

Case 3

A 27-year-old woman presented to the outpatient clinic with complaints of vaginal discharge. She also had irregular and heavy menstrual bleeding for one year. The complaints started following a dilatation and curettage of a first-trimester spontaneous abortion. There were no complaints of immediate post-abortal complications. She has had two previous vaginal deliveries preceding the abortion six years and eight years back which were uneventful. Her previous cycles were regular, with average blood flow and no associated dysmenorrhea.

**Figure 9:**
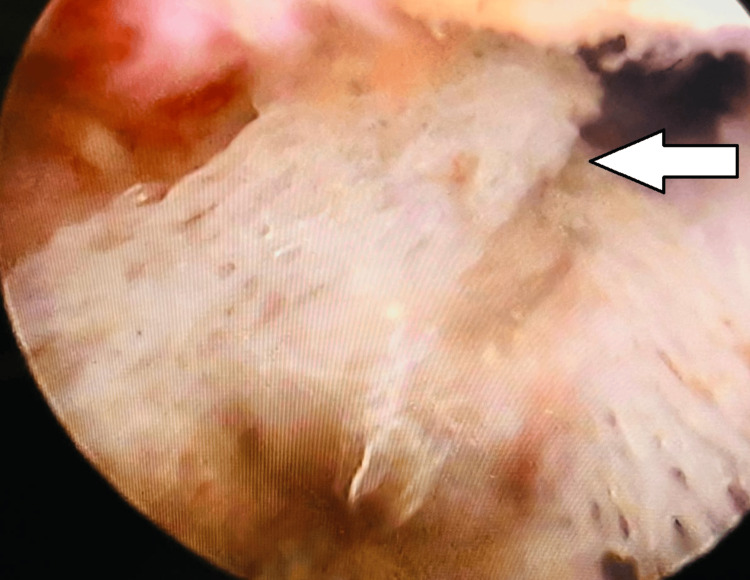
A hysteroscopic view of a trabecular bone fragment inside the uterine cavity (white arrow).

On examination, no significant finding was noted. On bimanual palpation, the uterus was of normal size and the adnexa was free. She underwent a transvaginal ultrasound which revealed an irregular endometrium with multiple echogenic foci, the largest measuring 15 mm (Figure 9).

A diagnostic hysteroscopy was performed. It showed numerous bone fragments inside the endometrial cavity. A 1.5-cm-long bony spicule was extracted using graspers and sent for histopathological examination. The histopathological findings were consistent with osseous metaplasia. The patient has subsequently resumed her normal menses and is symptom-free.

## Discussion

Two main theories have been described in the literature to explain the etiopathogenesis of osseous metaplasia of the endometrium: first is the retention of fetal bones secondary to previous abortions [4] and second is the metaplastic transformation of pluripotent stromal cells into osteoblasts secondary to chronic inflammation. In a nulliparous woman, the second hypothesis of metaplastic transformation is more obvious. However, in women with previous abortions, it is unclear if the bone formation occurs due to the metaplastic transformation of one's tissue or due to retained fetal tissue. Tulandi et al. found bones of fetal origin inside the uterine cavity, as confirmed by genetic analysis [4]. However, in a study performed by Cayuela et al., DNA analysis of the affected woman was compared to that of osseous tissue, and the genetic origin of both was identical [5]. Thus, the literature has conflicting evidence regarding the actual origin of the bone tissue.

In our study, case 1 is relevant in this regard, as we performed QF-PCR to compare the genetic makeup of the bone with that of the patient. Even though she had previous abortions, the DNA sample of the bone and patient was a complete match. Another differential is dystrophic calcification, defined as calcium deposition in devitalized and necrotic tissue [6]. It has been known to occur in the musculoskeletal system secondary to injury [6]. However, in this condition, there are no osteoblasts and the presence of purely calcium deposition. In our cases, the presence of osteoblasts excluded this possibility. It is interesting to know that metabolic disorders like hypervitaminosis D, hypercalcemia, and hyperphosphatemia are known to cause soft tissue calcifications [3]. In our study, all three cases did not have such metabolic disorders.

Women with osseous metaplasia of the endometrium present with vague gynecological symptoms and infertility. In a retrospective observational study, 63 women diagnosed with osseous metaplasia on hysteroscopy were included. It was found that dysmenorrhea, abnormal uterine bleeding, infertility, and at least one miscarriage were present in 34.9%, 27%, 23.8%, and 65.1%, respectively [7]. Secondary infertility may occur as the bone tissue causes local inflammation by the release of prostaglandins and has an intrauterine copper device (IUCD)-like effect. An interesting study found a reduction in menstrual flow and menstrual blood prostaglandin levels after the extraction of osseous metaplasia [8]. In our study, women in case 1 and case 3 presented with vaginal discharge and irregular vaginal bleeding (Table 1).

**Table 1:** Summary of the clinical features of the three cases of osseous metaplasia. TVS: transvaginal ultrasound; QF-PCR: quantitative fluorescence polymerase chain reaction; HPE: histopathological examination; ZN: Ziehl-Neelsen; AFB: acid-fast bacilli; MGIT: Mycobacteria Growth Indicator Tube; MRI: magnetic resonance imaging; STIR: short tau inversion recovery

Case	Parity	Previous abortions	Infertility	Symptoms	Imaging	Hysteroscopy findings	Additional investigations
1	P3L3A2	Yes	No	Irregular bleeding and vaginal discharge	TVS: multiple echogenic structures inside the endometrial cavity, the largest measuring 17.5 mm	Multiple bone-like structures inside the uterine cavity	QF-PCR of the bone and mother showed a 100% DNA match
2	Nulliparous	No	Yes	Secondary amenorrhea	TVS: a large collection with homogenous internal echoes and echogenic foci in the endometrial cavity. MRI showed a bulky uterus with well-defined T1 and T2/STIR hyperintense to intermediate signal intensity lesions in the lower uterine segment, endometrial cavity, and endocervical canal	100 ml of hematometra evacuated. Multiple intrauterine synechiae and multiple trabecular bony fragments in the endometrial cavity	Endometrial biopsy: ZN stain: no; AFB MGIT culture: sterile
3	P2L2A1	Yes	No	Menstrual abnormalities and vaginal discharge	TVS: irregular endometrium with multiple echogenic foci, the largest measuring 15 mm	1.5-cm-long bony spicule	None

Both women had previous surgical abortions which is consistent with the current evidence regarding the presentation of women with osseous metaplasia [7]. The second case was unique as the woman presented with secondary amenorrhea following a previous attempt at bone removal. On hysteroscopy, hematometra and intrauterine synechiae were found. The formation of hematomata secondary to osseous metaplasia was first reported in a patient who underwent a loop electrosurgical excision procedure (LEEP) for high-grade cervical intraepithelial neoplasia [9]. The author hypothesized that LEEP caused a local inflammatory state in the endometrium, promoting bone formation [9]. The pathophysiology behind osseous metaplasia is very interesting and is the key to further explaining the possible cause of synechiae formation and adhesions. Many authors suggest that osseous metaplasia develops secondary to differentiating totipotent stromal cells like fibroblasts into osteoblasts [3]. This change may be initiated by a chronic inflammatory cascade secondary to local trauma, intrauterine infections, and cervical intraepithelial neoplasia [10]. Thus, chronic inflammation is the key event behind the metaplastic transformation of non-osseous to osseous tissue. It has been stated that an interplay between various pro-inflammatory mediators mediates bone formation and resorption [11]. IL-12, IL-18, IL-33, and interferons (IFN) suppress osteoclastic differentiation and thus inhibit bone loss [11]. 

In case 2, one of the following can be a possible pathophysiology and explanation behind bone formation and intrauterine adhesions. First, previous intrauterine infection might have caused a pro-inflammatory milieu inside the uterine cavity promoting bone formation. Previous attempts to remove the bones could have further accelerated the local injury, inflammation, and repair cycle leading to the formation of intrauterine adhesions. Second, the intrauterine bone fragments in the dependent part of the uterine cavity might have caused mechanical obstruction leading to the formation of hematometra. Third, retained bones in the cavity might have undergone secondary infection leading to the release of inflammatory cytokines and adhesion formation. 

Transvaginal ultrasound is the first line of investigation to diagnose osseous metaplasia. The presence of echogenic foci with posterior acoustic shadowing is pathognomonic in this condition [11]. The coronal view of 3D ultrasound is useful in identifying irregular margins and differentiating them from other pathologies that come to mind like a retained IUCD or a foreign body [12]. In case of a further diagnostic dilemma, MRI can be used as an adjunct, in which other coexistent pathologies like hematometra and malignancies can also be evaluated. In case 1 and case 3, we did a 2D ultrasound only, before proceeding for hysteroscopy. However, in case 2, fluid collection inside the uterine cavity and echogenic foci found on 2D ultrasound raised suspicion for a hematometra. Therefore, we opted for an MRI.

The mainstay of management is the removal of the bone fragments under hysteroscopic view. This technique is useful for directly visualizing the tissue and ruling out other coexistent issues like intrauterine adhesions, endometritis, and malignancy. Hysteroscopy effectively relieves symptoms and leads to a return of fertility [8].

Our case series is unique as it is the third of its kind in this regard [4,5]. It highlights the maternal origin of osseous metaplasia by performing a genetic analysis. In addition, it sheds light on the fact that osseous metaplasia may present with secondary amenorrhea and intrauterine synechiae, which is a unique presentation. However, our study is not without limitations. Firstly, we couldn't do genetic testing in all three cases as the patient did not consent to the same due to financial constraints. Secondly, because of the low number of cases, it is early to state the existing correlation between patient symptoms and osseous metaplasia. Larger studies need to be performed to support these findings. Thirdly, the cause-effect relationship between osseous metaplasia and intrauterine adhesions is doubtful, and further studies are necessary to throw light in this direction. Further research is needed to understand the link between chronic inflammation and the development of osseous metaplasia. Other future research prospects include investigating new diagnostic tools like noninvasive biomarkers in blood or menstrual blood, which may help in the diagnosis and monitoring of such cases.

## Conclusions

Osseous metaplasia of the endometrium is a rare entity that may have a varied presentation. Its primary differential is the retention of fetal bones secondary to previous abortion. The genetic origin of bones in the uterine cavity can be confirmed by genetic testing. In our study, QF-PCR testing of the bone was done, suggesting that it is genetically derived from the patient and not from the fetus.
